# P371: Utilization of alternating currents as a novel procedure for increasing of disinfection efficacy against Staphylococcus aureus and Pseudomonas aeroginosa

**DOI:** 10.1186/2047-2994-2-S1-P371

**Published:** 2013-06-20

**Authors:** M Nasiri, M Mirzaii, A Alfi, P Norozi, F Davar Doost, M Fazli

**Affiliations:** 1 Shahroud University of Medical Sciences, Shahroud, Iran, Islamic Republic Of; 2 Shahroud University of Technology, Shahroud, Iran, Islamic Republic Of; 3 Azad Damghan University, Shahroud, Iran, Islamic Republic Of; 4 Azad Shahroud University, Shahroud, Iran, Islamic Republic Of

## Introduction

The use of physical means as an aid for modern medicine in the champion against pathogenic microorganisms holds new approach that recently have begun to be widely recognized. The use of an additional physical means, alternating currents, introduced to inhibit bacterial growth and enhance disinfectant potency.The purposes of the present study were (i) to find out the best frequency of alternating currents can inhibit the growth of bacteria and (ii) to determine efficacy alternating currents on disinfectant bactericidal potency.

## Methods

Electric field strength of 12 and 20 V/cm at 50KHz, 10MHz, 20 MHz was applied continuously during course of staphylococcal and pseudomonas lag phase. Then Changes in growth of bacteria investigated by time kill method. Efficacy alternating currents on current disinfectants bactericidal potency (microzed, deconex, dettol glutaraldehyde) evaluated by MIC and MBC.

## Results

The best bacteriostatic effect showed due to electric field strength of 10 V/cm at 20MHz(s. aureus and p. aeroginosa decreased 1.1 log and 1 log respectively).Effectiveness of disinfectants electric field strength of 10V/cm at 20MHz on low and average performance (phenolic compounds and ammonia) than the high-level disinfectants (glutaraldehyde dialdehydes) was.

## Conclusion

It is necessary to find out suitable alternating current form in future. This method might be applied as a complementary to eliminate pollution of waters and increase disinfectant bactericidal potency.

## Disclosure of interest

None declared

